# EIF4A3-induced circular RNA MMP9 (circMMP9) acts as a sponge of miR-124 and promotes glioblastoma multiforme cell tumorigenesis

**DOI:** 10.1186/s12943-018-0911-0

**Published:** 2018-11-23

**Authors:** Renjie Wang, Sai Zhang, Xuyi Chen, Nan Li, Jianwei Li, Ruichao Jia, Yuanqing Pan, Haiqian Liang

**Affiliations:** 1 Institute of Traumatic Brain Injury and Neurology, Characteristic Medical Center of Chinese People’s Armed Police Force, Tianjin, 300162 China; 2 Department of Neurosurgery, Characteristic Medical Center of Chinese People’s Armed Police Force, Tianjin, 300162 China; 3 Department of Basic Medicine, Tianjin Medical College, Tianjin, 300222 China; 4 Chinese Glioma Cooperative Group (CGCG), Tianjin, China

**Keywords:** Circular RNA MMP9, Eukaryotic initiation factor 4A3, microRNA-124, Glioblastoma multiforme, Migration and invasion

## Abstract

**Background:**

Circular RNAs (circRNAs) have been found to play critical roles in the development and progression of various cancers. However, little is known about the effects of the circular RNA network on glioblastoma multiforme (GBM).

**Methods:**

A microarray was used to screen circRNA expression in GBM. Quantitative real-time PCR was used to detect the expression of circMMP9. GBM cells were transfected with a circMMP9 overexpression vector or siRNA, and cell proliferation, migration and invasion, as well as tumorigenesis in nude mice, were assessed to examine the effect of circMMP9 in GBM. Biotin-coupled miRNA capture, fluorescence in situ hybridization and luciferase reporter assays were conducted to confirm the relationship between circMMP9 and miR-124.

**Results:**

In this study, we screened differentially expressed circRNAs and identified circMMP9 in GBM. We found that circMMP9 acted as an oncogene, was upregulated in GBM and promoted the proliferation, migration and invasion abilities of GBM cells. Next, we verified that circMMP9 served as a sponge that directly targeted miR-124; circMMP9 accelerated GBM cell proliferation, migration and invasion by targeting miR-124. Furthermore, we found that cyclin-dependent kinase 4 (CDK4) and aurora kinase A (AURKA) were involved in circMMP9/miR-124 axis-induced GBM tumorigenesis. Finally, we found that eukaryotic initiation factor 4A3 (eIF4A3), which binds to the MMP9 mRNA transcript, induced circMMP9 cyclization and increased circMMP9 expression in GBM.

**Conclusions:**

Our findings indicate that eIF4A3-induced circMMP9 is an important underlying mechanism in GBM cell proliferation, invasion and metastasis through modulation of the miR-124 signaling pathway, which could provide pivotal potential therapeutic targets for the treatment of GBM.

**Graphical abstract:**

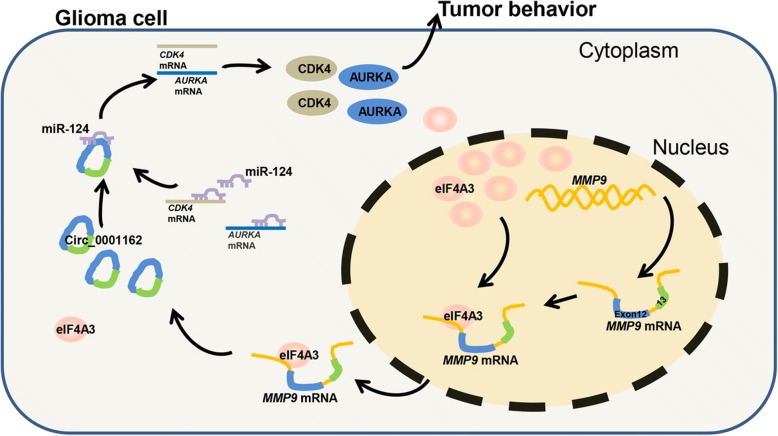

**Electronic supplementary material:**

The online version of this article (10.1186/s12943-018-0911-0) contains supplementary material, which is available to authorized users.

## Background

Glioblastoma multiforme (GBM), also known as grade IV glioma, is the most aggressive glioma [1, 2]. Despite the progress in comprehensive therapeutic strategies, including surgery, radiation and chemotherapy, the median survival of patients has barely improved and remains only approximately 14 months [3, 4]. Thus, effective preventative and therapeutic measures are urgently needed for patients with GBM.

Circular RNAs (circRNAs) are a new type of noncoding RNA (ncRNA) with a closed continuous loop structure and have become a new focus of study despite being discovered 20 years ago [5]. They are a class of endogenous noncoding RNAs without a 5′ end cap or a 3′ terminal poly (A) tail structure [6]. Compared with linear RNA, circRNA is more stable and is not affected by RNA exonucleases, deadenylation or cap removal [7]. Multiple studies have shown that circRNAs can regulate genes and exhibit tissue specificity in humans [8–10]. Studies have also confirmed that circRNAs participate in various biological or pathological processes, such as proliferation, apoptosis, migration and invasion [11–13]. With the development of high-throughput sequencing and novel computational technology, many previously unknown circRNAs have been verified to play a role in various diseases [8, 14–16]. However, the mechanisms and functions of circRNAs are not fully clear in GBM. In the present study, we aimed to identify circRNAs that may be involved in the pathology of GBM using microarray. In addition, we screened and identified the expression and functions of hsa_circ_0001162 (circMMP9) derived from matrix metalloproteinase-9 (MMP-9) in GBM and examined the detailed mechanism of this circRNA in GBM progression.

Competitive endogenous RNAs (ceRNAs), which compete for the shared miRNA response elements (MREs) of miRNAs, can affect the expression of miRNA target genes [17, 18]. Previous studies have claimed that lncRNAs, circRNAs and pseudogenes can serve as miRNA “sponges” by sharing common MREs to regulate gene expression [19–21]. Presently, the ceRNA regulation model has become an important mechanism in various cancers [22–24]. In the present study, we designed a series of functional and molecular assays to explore the ceRNA mechanism of circMMP9 and found that cyclin-dependent kinase 4 (CDK4) and aurora kinase A (AURKA) constitute a ceRNA regulation network for the circMMP9/miR-124 axis in GBM.

## Methods

### Clinical samples

GBM tissues and corresponding normal tissues (2 cm away from the tumor) were obtained from 18 patients who were diagnosed with GBM at Characteristic Medical Center of Chinese People's Armed Police Force from March 2014 to September 2017. The detailed clinical patient information is shown in **(**Additional file 1: Table S1**)**. Approval for this study was provided by the Ethics Committee of Characteristic Medical Center of Chinese People's Armed Police Force. Informed consent was obtained from each GBM patient. Tissues were immersed in liquid nitrogen immediately after removal from patients and were stored at − 80 °C until use.

### RNA extraction and quantitative real-time PCR (qRT-PCR)

Total RNA was extracted from GBM tissues, corresponding normal tissues and treated U87 and U251 cells using TRIzol reagent (Invitrogen; Thermo Fisher Scientific, Inc.) according to the manufacturer’s protocol. A High-Capacity cDNA Reverse Transcription kit (Applied Biosystems, Foster City, CA, USA) was used to synthesize cDNA from RNA according to the manufacturer’s protocol. qRT-PCR was performed using a SYBR Green kit (Bio-Rad Laboratories, Inc., Hercules, CA, USA) in an ABI 7900 PCR Thermal Cycler according to the manufacturer’s protocol. The mRNA expression levels of genes were measured using the 2^-△△Ct^ method [25]. Small RNA RNU6 (U6) was used as the endogenous control to normalize miRNA expression, while GAPDH was used as the endogenous control to normalize circRNA expression.

### Microarray

Total RNA was extracted and purified using the mirVana™ miRNA Isolation Kit (cat.# AM1561; Ambion, Austin, TX, USA) following the manufacturer’s instructions and was evaluated for an RIN number to assess RNA integration using an Agilent Bioanalyzer 2100 (Agilent Technologies, Santa Clara, CA, USA). Total RNA was amplified and labeled using the Low Input Quick Amp WT Labeling Kit (cat.# 5190–2943; Agilent Technologies). Labeled cRNA was purified using the RNeasy mini kit (cat.# 74,106; Qiagen, Hilden, Germany).

Each slide was hybridized with 1.65 μg of Cy3-labeled cRNA using the Gene Expression Hybridization Kit (cat.# 5188–5242; Agilent Technologies) in a hybridization oven (cat.# G2545A; Agilent Technologies). After 17 h of hybridization, the slides were washed in staining dishes (cat.# 121; Thermo Scientific, Waltham, MA, USA) using the Gene Expression Wash Buffer Kit (cat.# 5188–5327; Agilent Technologies).

The slides were scanned using the Agilent Microarray Scanner (cat.#G2565CA; Agilent Technologies) using the default settings: dye channel, green; scan resolution, 3 μm; PMT, 100%; 20 bits. The data were extracted using Feature Extraction software 10.7 (Agilent Technologies). Raw data were normalized using the Quantile algorithm in the limma package in R. Microarray analysis was performed by Shanghai Biotechnology Cooperation (Shanghai, P.R. China).

### Pull-down assay

MMP9 expression vectors, which were truncated to varying degrees (a1-a5), were constructed. A biotinylated MMP9 DNA probe was designed and synthesized by GenePharma Co., Ltd. (Shanghai, China). According to the protocol of the manufacturer, probe-coated beads were produced by dissolving the probe and incubating them with Dynabeads M-280 Streptavidin (Invitrogen, CA, USA) for 10 min at room temperature. U87 cell lysates were treated with the probe-coated beads, and the results were detected by qRT-PCR analysis.

### RNA binding protein immunoprecipitation (RIP) assay

According to the manufacturer’s protocol, the RIP assay was performed using an EZMagna RIP kit (Millipore, Billerica, MA, USA). U87 cells were collected and lysed with RIP lysis buffer. U87 cell lysates (100 μl) were treated with RIP buffer and were incubated with Proteinase K and magnetic beads conjugated with anti- eIF4A3 antibody or control (IgG). Next, the immunoprecipitated RNA was extracted. The results were measured by qRT-PCR.

### Animal studies

Four-week-old male nude mice were purchased from the National Laboratory Animal Center (Shanghai, China). The animal studies were approved by the Institutional Animal Care and Use Committee of Pingjin Hospital. In total, 20 mice (*n* = 5 each group) were injected with the stable circMMP9 overexpressing U87 cells, circMMP9-knockdown U251 cells (1 × 10^6^) or parent control GBM cells resuspended in growth medium (150 μL) and Matrigel substrate (150 μL). Both the stable U87 and U251 cells contained a GFP marker. The mice were injected with 4.0 mg of luciferin (Gold Biotech) in 50 μl of saline. After 1 h, tumors were detected using an IVIS@ Lumina II system (Caliper Life Sciences, Hopkinton, MA). The animals were sacrificed 28 days after injection, and the tumors were collected to measure the tumor volume every 7 days. The tumor volume was calculated using the following formula: volume (mm^3^) = length × width^2^/2.

### Statistical analysis

The data were analyzed using Student’s t-test and ANOVA using SPSS 15.0 software (SPSS, Chicago, IL, USA). Each experiment was repeated at least three times. All results were summarized and are presented as means ± standard deviation (SD). A *P* value less than 0.05 was considered statistically significant. Additional Supplementary Materials and Methods, including FISH and microarray analyses, are described in the supplementary files.

## Results

### Identification of circMMP9 in GBM via microarray analysis

**Fig. 1:**
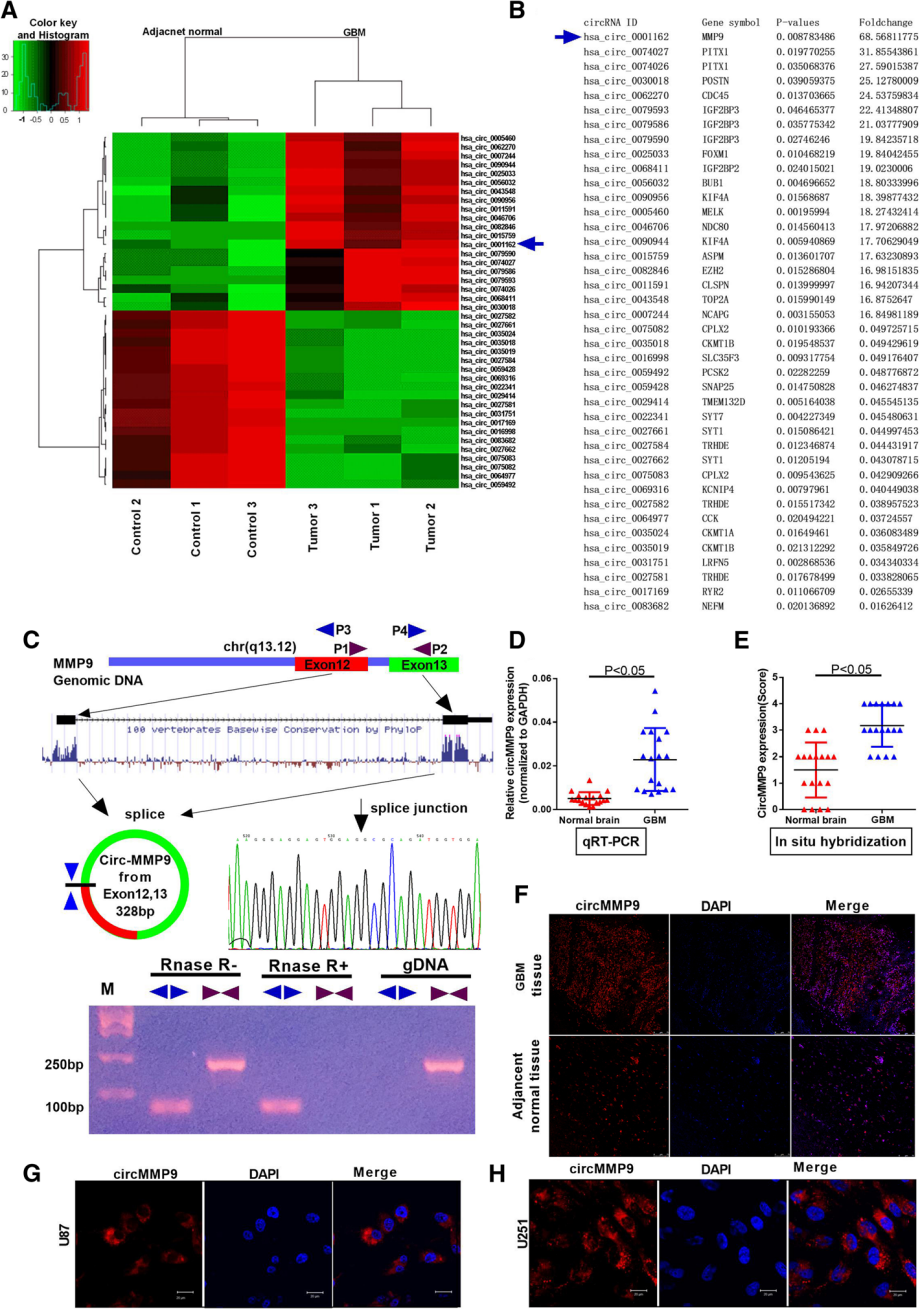
Characterization of circMMP9 in human GBM. a Clustered heat map showing tissue-specific circRNAs (top 20 upregulated and downregulated circRNAs), which are displayed on a scale from green (low) to red (high), between three human GBM tissues and adjacent normal tissues. The arrow represents the circRNA (hsa_circ_001162) with the greatest differential expression. b Detailed information for the top 20 upregulated and downregulated circRNAs according to the extent. c Schematic representation of circMMP9 formation. The splice junction sequence was Sanger sequenced, and the RNAs were detected via PCR. Divergent primers could produce circRNAs in cDNA but not in genomic DNA (gDNA); convergent primers could produce cDNA and gDNA. d The expression level of circMMP9 was detected by qRT-PCR in GBM tissues and adjacent normal brain tissues (*n* = 18, *P* < 0.05); GAPDH served as the internal control. **e-f** circMMP9 expression was measured using in situ hybridization (FISH) in GBM tissues and adjacent normal brain tissues (*n* = 18, *P* < 0.05). **g-h** Confocal FISH was performed to determine the location of circMMP9 in U87 and U251 cells

We selected 3 paired GBM tissues to carry out the microarray assay to determine the expression status of circRNAs in GBM tissues. Next, the host genes of differentially expressed circRNAs were subjected to GO analysis **(**Additional file 2: Figure S1A and S1B**)**. The clustered heat map in Fig. 1a shows the top 20 upregulated and downregulated circRNAs. hsa_circ_0001162 (circMMP9) was the circRNA with the greatest differential expression (Fig. 1b). Using a bioinformatics method (UCSC Date), we then explored circMMP9 formation and found that circMMP9, with a molecular weight of 328 bp, was formed from exons 12 and 13 of MMP9 (Fig. 1c). PCR analysis indicated that divergent primers could produce the circular isoform of MMP9 with cDNA but not with genomic DNA (gDNA), while convergent primers could amplify the linear isoform of MMP9 from both cDNA and gDNA in the 3 GBM tissues, U87 cells and U251 cells (Additional file 2: Figure S1C). Furthermore, qRT-PCR showed that circMMP9 can resist RNase R, while MMP9 mRNA can be degraded by RNase R (Fig. 1c, Additional file 2: Figure S1D). Sanger sequencing of the PCR products using divergent primers also confirmed the presence of a splice junction in circMMP9 (Fig. 1c). To explore the circMMP9 expression level in GBM, we used a qRT-PCR assay to measure its expression in 18 pairs of GBM and adjacent normal brain tissues. The results indicated that the expression level of circMMP9 was significantly increased in GBM tissues compared with that in normal brain tissues (*P* < 0.05, Fig. 1d). FISH assay results also showed that circMMP9 was highly expressed in GBM tissues (*P* < 0.05, Fig. 1e-f). In addition, we explored the location of circMMP9 in U87 and U251 cells. Confocal FISH assay results revealed that circMMP9 was primarily expressed in the cytoplasm (Fig. 1g-h). The above results suggest that circMMP9 may play important roles in GBM pathology.

### circMMP9 is oncogenic and associated with proliferation, migration and invasion in GBM cells

**Fig. 2:**
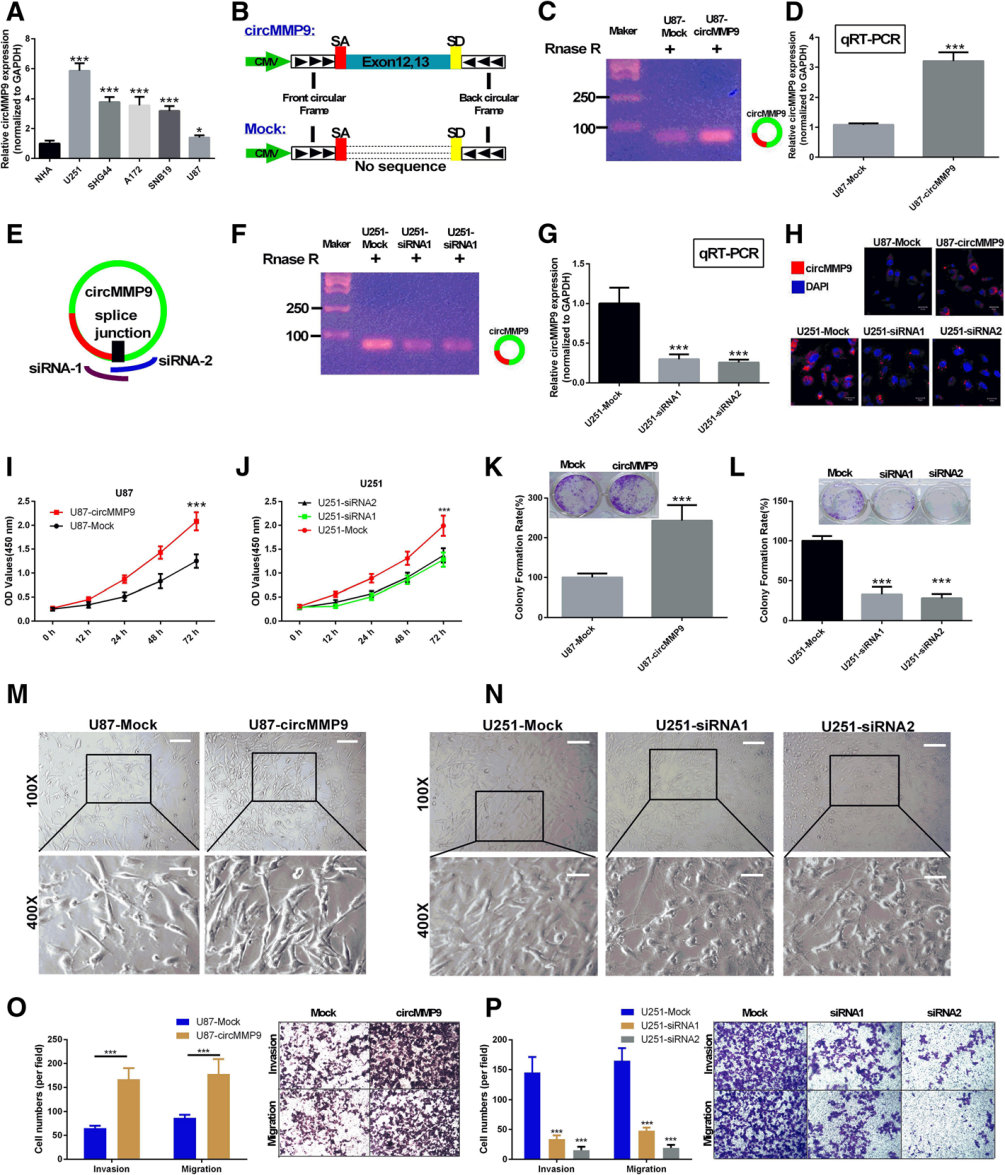
circMMP9 is oncogenic and associated with proliferation, migration and invasion in GBM cells. **a** circMMP9 expression was analyzed by qRT-PCR in normal human astrocytes (NHAs) and GBM cells (U251, SHG44, A172, SNB19 and U87) (*P < 0.05, ***P < 0.001). **b** Schematic representation of circMMP9 and mock plasmid construction. **c** circMMP9 expression was measured by PCR in the circMMP9 plasmid or mock plasmid-transfected U87 cells using RNase R treatment. **d** qRT-PCR was performed to detect circMMP9 expression in treated U87 cells (***P < 0.001). **e** Schematic representation of designed siRNAs (siRNA1 and siRNA2) for circMMP9 at splice junctions. **f** circMMP9 expression was detected by PCR in circMMP9 siRNA1, siRNA2 or mock-transfected U251 cells with RNase R treatment. **g** qRT-PCR was performed to analyze circMMP9 expression in treated U251 cells (***P < 0.001). **h** circMMP9 expression was measured using FISH in transfected U87 and U251 cells. Red represents the circMMP9 probe, while DAPI was used to stain cell nuclei. **i-l** Cell proliferation abilities were detected by CCK-8 and colony formation assays in transfected U87 and U251 cells (***P < 0.001). **m-n** Cell morphology was observed under a microscope. Magnification, 100×, 400×. (**O-P**) Cell migration and invasion abilities were measured with Transwell assays (***P < 0.001)

First, we detected the expression of circMMP9 in normal human astrocytes (NHAs) and GBM cells (U251, SHG44, A172, SNB19 and U87). We found that circMMP9 was highly expressed in U251 cells and was poorly expressed in U87 cells (*P* < 0.05, *P* < 0.001, Fig. 2a). Therefore, U251 and U87 cells were chosen for further study. Next, circMMP9 and mock expression vectors were constructed (Fig. 2b). U87 cells were transfected with circMMP9 or mock plasmids, and the total RNA was then treated with RNase R. The results indicated that the circMMP9 overexpression vector was efficient (*P* < 0.001, Fig. 2c-d). Simultaneously, we analyzed the sequences of siRNAs for circMMP9 at splice junctions and synthesized circMMP9 siRNA1 and siRNA2 (Fig. 2e). U251 cells were transfected with circMMP9 siRNA1, siRNA2 or mock for 48 h. The results revealed that circMMP9 expression was knocked down by siRNA1 or siRNA2 compared with that in the mock group (*P* < 0.001, Fig. 2f-g); FISH assay verified these results (Fig. 2h). Furthermore, we confirmed that overexpression of circMMP9 significantly promoted the proliferation ability of U87 cells (*P* < 0.001, Fig. 2i and k). Silencing of circMMP9 significantly inhibited the proliferation ability of U251 cells (*P* < 0.001, Fig. 2j and l). Next, we observed the influence of circMMP9 on GBM cell morphology and found that cells were spindle-shaped, irregular and in a disordered arrangement in the U87-circMMP9 group (Fig. 2m) and were relatively single, short shuttle-like or round in the U251-siRNA1 and U251-siRNA2 groups (Fig. 2n). We also found that overexpression of circMMP9 significantly promoted the migration and invasion abilities of U87 cells (*P* < 0.001, Fig. 2o). Silencing of circMMP9 significantly inhibited the migration and invasion abilities of U251 cells (*P* < 0.001, Fig. 2p).

### circMMP9 acts as a sponge and directly targets miR-124

**Fig. 3:**
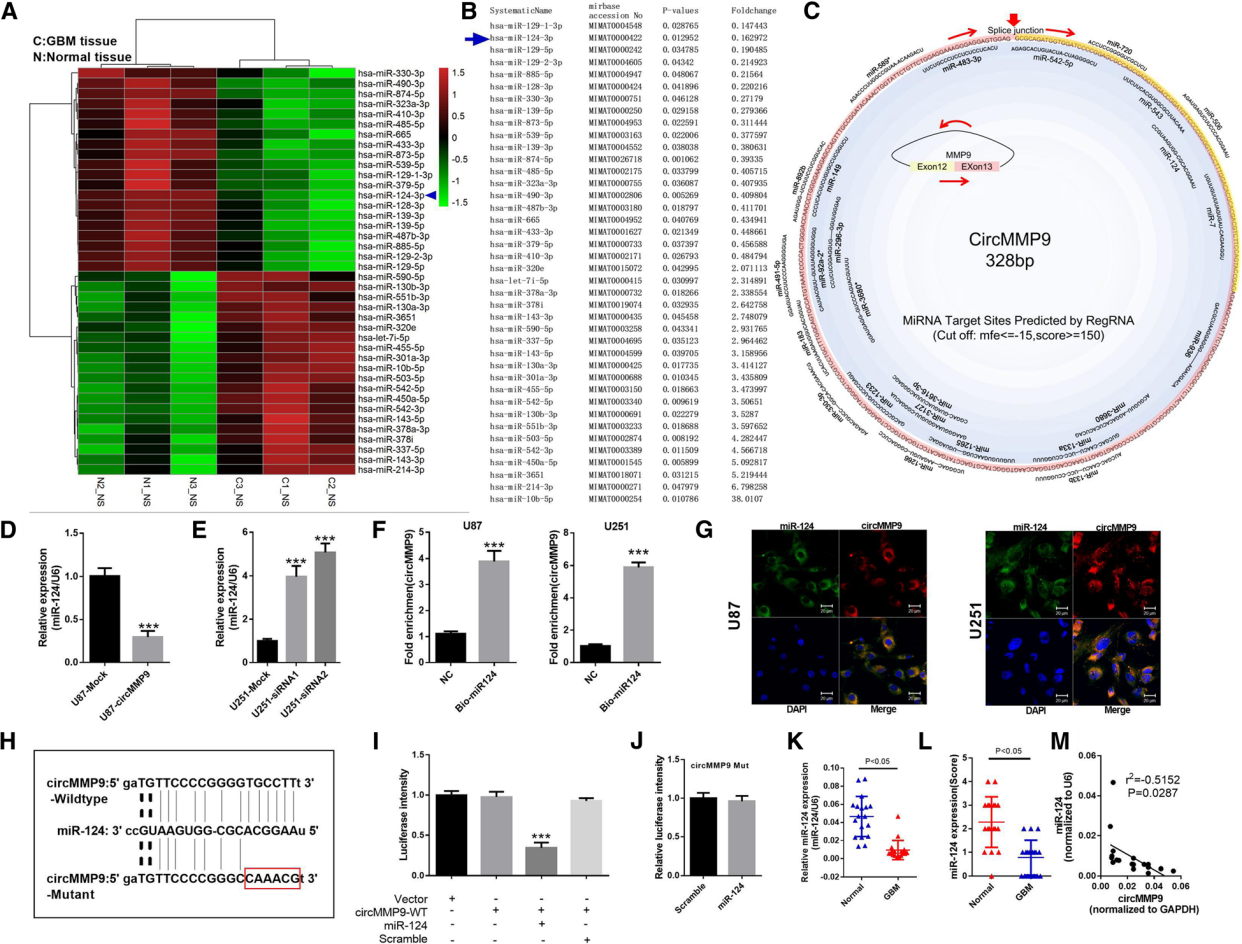
circMMP9 acts as a sponge of miR-124. **a** Profile of the top 20 upregulated and downregulated miRNAs in human adjacent normal tissues and GBM tissues. Green indicates low expression, and red indicates high expression. The arrow represents miR-124-3p (miR-124). **b** Detailed information for the top 20 upregulated and downregulated miRNAs according to the extent. **c** The binding sites of miRNAs and circMMP9 were predicted by RegRNA (http://regrna.mbc.nctu.edu.tw/). **d-e** miR-124 expression was detected by qRT-PCR in treated U87 or U251 cells (***P < 0.001). **f** Binding of circMMP9 and miR-124 was analyzed using the pull-down assay (***P < 0.001). **g** Colocalization of circMMP9 and miR-124 was measured using FISH in transfected U87 and U251 cells. **h** The putative binding sites of miR-124 on the circMMP9 wild-type (WT) or mutated sequence are shown. **i** A luciferase reporter assay was performed to detect the activity of circMMP9 in U87 cells cotransfected with miR-124 or scramble and circMMP9 or vector (***P < 0.001). (**J**) U87 cells were cotransfected with miR-124 or scramble and mutated circMMP9, and the activity of circMMP9 was detected using the dual-luciferase reporter assay after transfection. **k** miR-124 expression was detected by qRT-PCR in GBM tissues and normal brain tissues (n = 18, *P* < 0.05). **l** miR-124 expression was measured via IHC in GBM tissues and normal brain tissues (*n* = 18, *P* < 0.05). **m** Correlation between the expression of miR-124 and circMMP9 was evaluated by Pearson’s correlation test (r^2^ = − 0.5152, *P* = 0.0287)

The previous results indicated that circMMP9 was primarily expressed in the cytoplasm, indicating that circMMP9 may serve as a miRNA sponge in GBM. To identify the downstream miRNAs of circMMP9, a miRNA microarray was performed in the same GBM tissues. In Fig. 3a, the top 20 upregulated and downregulated miRNAs are shown. Based on the fold change and *P* value, we found that hsa-miR-129-1-3p, hsa-miR-124-3p and hsa-miR-129-5p were the three miRNAs with the greatest differential expression (Fig. 3b). Using bioinformatics analysis (RegRNA prediction, with a cutoff: mfe ≤ − 15, score ≥ 150), we found that miR-124 had a putative binding site with circMMP9 (Fig. 3c). These results indicated that circMMP9 may serve as a sponge of miR-124 in GBM. Indeed, our results showed that overexpression of circMMP9 in U87 cells markedly decreased miR-124 expression, while the FISH assay suggested that circMMP9 and miR-124 were colocalized in the cytoplasm of U87 cells (*P* < 0.001, Fig. 3 d and g); silencing of circMMP9 in U251 cells markedly increased miR-124 expression, while the FISH assay suggested that circMMP9 and miR-124 were colocalized in the cytoplasm of U87 cells (*P* < 0.001, Fig. 3e and g). Pull-down assay results also indicated that circMMP9 was enriched in the biotin-labeled miR-124 group (*P* < 0.001, Fig. 3f). Additionally, a dual-luciferase reporter assay was performed to confirm the interaction between circMMP9 and miR-124, and the data revealed that transfection with an miR-124 mimic observably attenuated the luciferase activity of wild-type (WT) circMMP9 compared with the scrambled control (*P* < 0.001, Fig. 3h and i). Meanwhile, the miR-124 mimic did not affect the luciferase activity of circMMP9-mut (Fig. 3j). Subsequently, we evaluated the expression level of miR-124 in GBM tissues, and the results showed that miR-124 was downregulated in GBM tissues compared with that in adjacent normal tissues (*P* < 0.05, Fig. 3k-l). There was also a negative correlation between circMMP9 and miR-124 (r^2^ = − 0.5152, *P* = 0.0287, Fig. 3m).

### circMMP9 accelerates GBM cell proliferation, migration and invasion by targeting miR-124

**Fig. 4:**
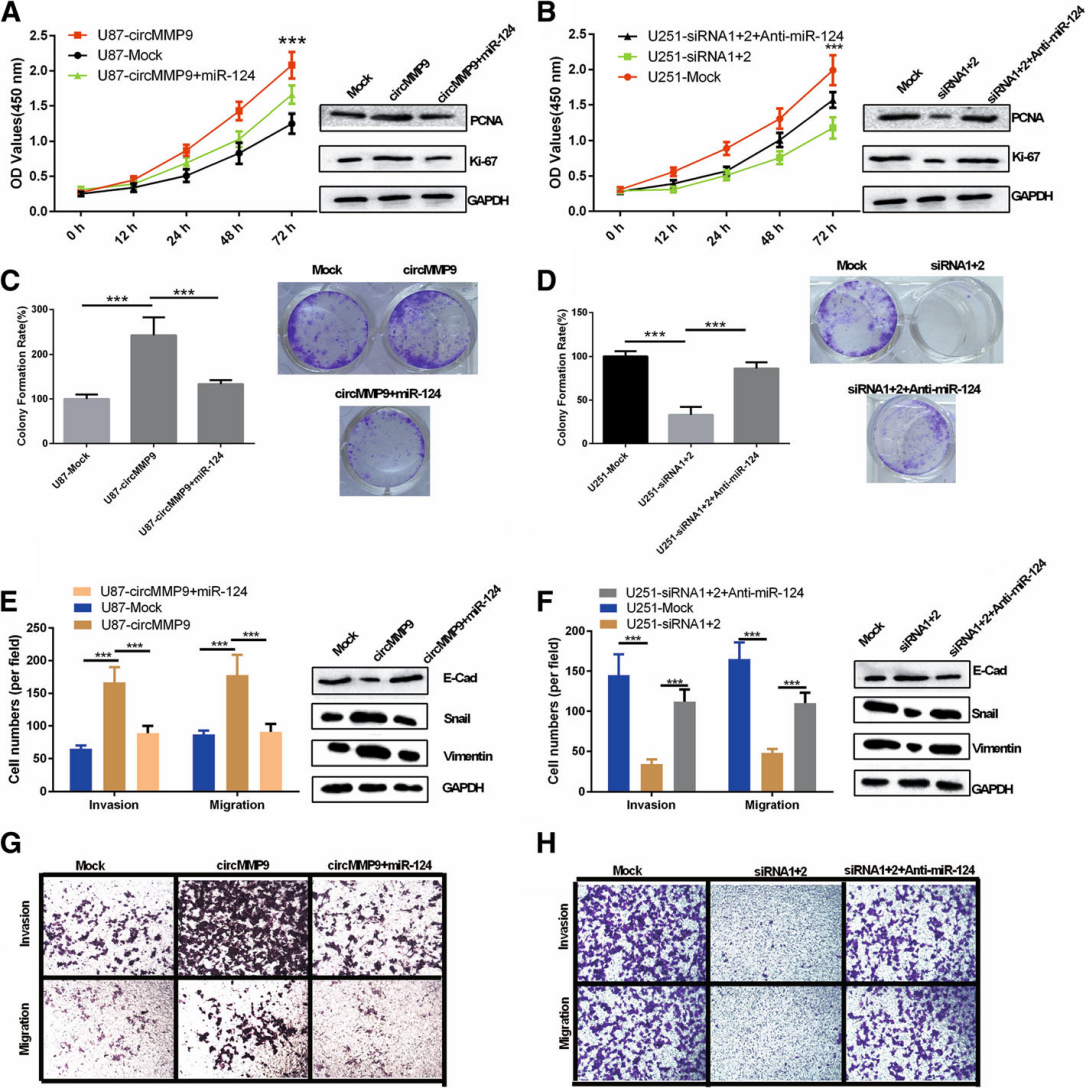
circMMP9 accelerates GBM cell proliferation, migration and invasion by targeting miR-124. **a-d** U87 cells were transfected with mock, circMMP9 plasmid, or circMMP9 plasmid and miR-124; U251 cells were transfected with mock, circMMP9 siRNA1 + 2, or circMMP9 siRNA1 + 2 and anti-miR-124. CCK-8 and colony formation assays were performed to assess the proliferation ability of the transfected U87 and U251 cells (***P < 0.001). Western blot assays were used to analyze the protein expression levels of PCNA and Ki67 in transfected U87 and U251 cells. **e-h** Transwell assays were performed to evaluate cell migration and invasion abilities (***P < 0.001). Western blot assays were used to analyze the protein expression levels of E-cadherin (E-cad), snail and vimentin in transfected U87 and U251 cells

To further investigate the roles of miR-124 and circMMP9 in GBM progression, we performed rescue assays to evaluate the effects of the circMMP9/miR-124 axis on the proliferation, migration and invasion abilities of GBM cells. The results indicated that circMMP9 promoted U87 cell proliferation and miR-124 weakened this promotion. circMMP9 also increased the expression levels of proliferation-associated markers (PCNA and Ki-67), and miR-124 reversed this tendency (*P* < 0.001, Fig. 4a and c). Meanwhile, silencing of circMMP9 inhibited U251 cell proliferation, and an miR-124 inhibitor (anti-miR-124) attenuated this inhibition. Additionally, silencing of circMMP9 decreased the expression levels of proliferation-associated markers (PCNA and Ki-67), and anti-miR-124 reversed this tendency (*P* < 0.001, Fig. 4b and d). Furthermore, we also found that circMMP9 accelerated U87 cell migration and invasion and miR-124 weakened this acceleration (*P* < 0.001, Fig. 4e and g). Meanwhile, silencing of circMMP9 suppressed U251 cell migration and invasion, and anti-miR-124 attenuated this suppression (*P* < 0.001, Fig. 4f and h). Western blot assays revealed that the circMMP9/miR-124 axis regulated EMT marker (E-cadherin, snail and vimentin) expression in GBM cells (Fig. 4e-right side and Fig. 4f-right side).

### circMMP9 upregulates CDK4 and AURKA via miR-124

**Fig. 5:**
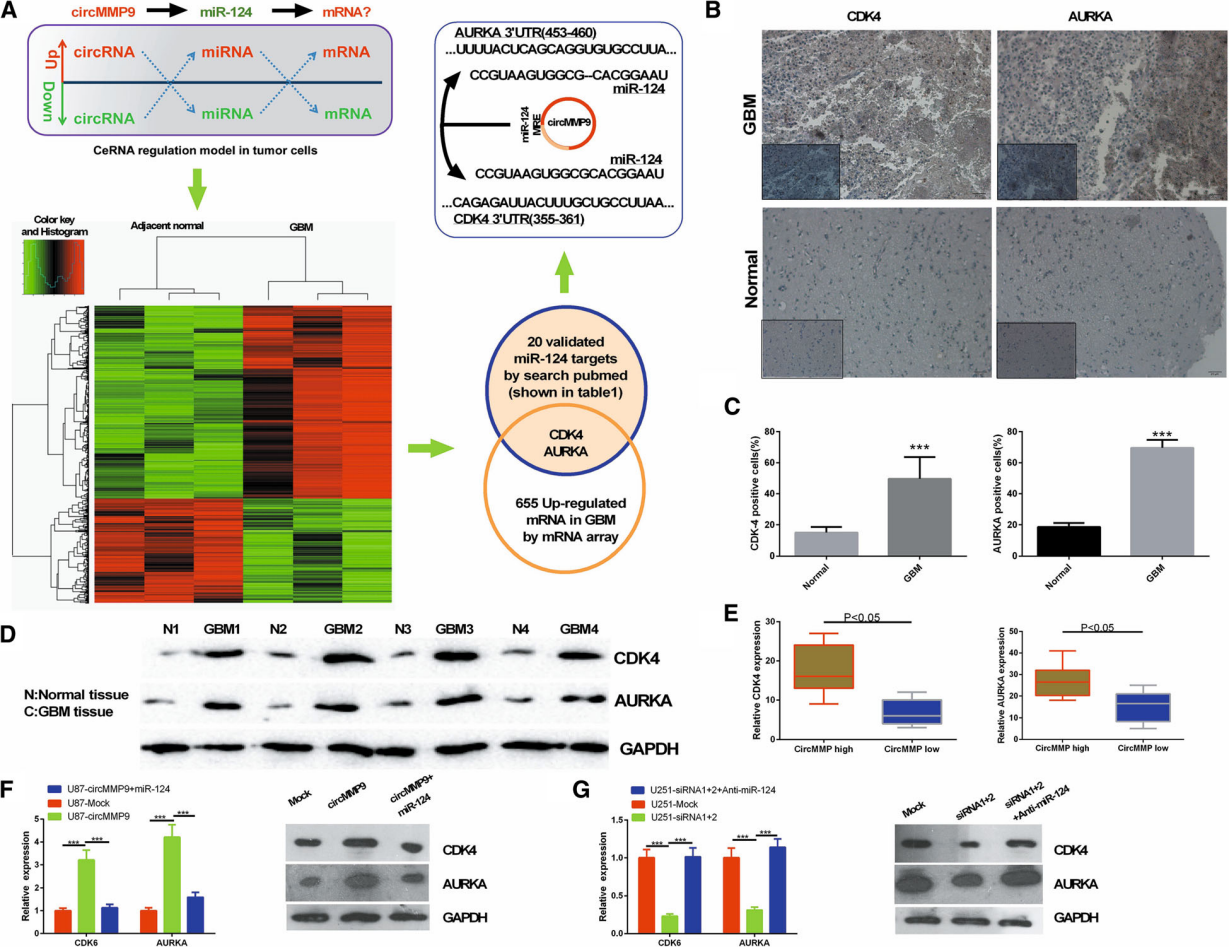
circMMP9 upregulates CDK4 and AURKA via miR-124. **a** Flow chart to screen the targets of miR-124 in GBM. **b** CDK4 and AURKA expression was detected via IHC in GBM tissues and adjacent normal tissues. **c** The cells positive for CDK4 and AURKA were counted (***P < 0.001). **d** The protein expression levels of CDK4 and AURKA were measured by western blot assays in GBM tissues and adjacent normal tissues. **e** CDK4 and AURKA expression was evaluated by qRT-PCR in GBM tissues with high circMMP9 expression or low circMMP9 expression (*P* < 0.05). **f** U87 cells were transfected with mock, circMMP9 plasmid, or circMMP9 plasmid and miR-124. CDK4 and AURKA expression was detected by qRT-PCR and western blot assays (***P < 0.001). **g** U251 cells were transfected with mock, circMMP9 siRNA1 + 2, circMMP9 siRNA1 + 2 or anti-miR-124. CDK4 and AURKA expression was detected by qRT-PCR and western blot assays (***P < 0.001)

Next, we further explored which signaling pathway might be affected by the circMMP9/miR-124 axis to regulate the GBM phenotype. According to the ceRNA regulation model in tumor cells, an mRNA array was performed to analyze the differentially expressed mRNAs in the original three paired GBM tissues. In total, 655 upregulated mRNAs were found in GBM tissues compared with that in the adjacent normal brain tissues. Additionally, we used PubMed to screen miR-124 target genes and found 20 validated targets in GBM (Table 1). By Venn analysis, we found that CDK4 and AURKA were the target genes of miR-124 and may be regulated by circMMP9 in GBM (Fig. 5a). First, we confirmed the expression of CDK4 and AURKA in four paired GBM tissues via IHC and western blotting, and the results indicated that they exhibited higher expression in GBM tissues (*P* < 0.001, Fig. 5b, c and d). Additionally, their expression had the same tendency as that of circMMP9 (*P* < 0.001, Fig. 5e). These results suggest that CDK4 and AURKA may serve as downstream factors in the circMMP9/miR-124 axis. Indeed, western blot assays revealed that circMMP9 significantly regulates CDK4 and AURKA expression via miR-124 (*P* < 0.001; Fig. 5f and g).

**Table 1:** MiR-124 target genes in glioma searched in Pubmed

Gene symbol	Gene full name	Location	PubMed ID
SMAD4	SMAD family member 4	Chromosome 18, NC_000018.10 (51,030,213..51085042)	28,791,348
MGMT	O-6-methylguanine-DNA methyltransferase	Chromosome 10, NC_000010.11 (129,467,184..129770983)	27,057,640
CAPN4	Calpain small subunit 1	Chromosome 19, NC_000019.10 (36,139,926..36150353)	26,530,859
CDK4	Cyclin dependent kinase 4	Chromosome 12, NC_000012.12 (57,747,727..57752447, complement)	23,761,023
IQGAP1	IQ motif containing GTPase activating protein 1	Chromosome 15, NC_000015.10 (90,388,241..90502243)	25,175,832
CTNNB1	Catenin beta 1	Chromosome 3, NC_000003.12 (41,199,451..41240448)	25,175,832
ROCK1	Rho associated coiled-coil containing protein kinase 1	Chromosome 18, NC_000018.10 (20,949,740..21111851, complement)	23,936,026
SOS1	SOS Ras/Rac guanine nucleotide exchange factor 1	Chromosome 2, NC_000002.12 (38,981,549..39124959, complement)	23,817,964
CLOCK	Clock circadian regulator	Chromosome 4, NC_000004.12 (55,427,901..55547138, complement)	23,792,158
PPP1R13L	protein phosphatase 1 regulatory subunit 13 like	Chromosome 19, NC_000019.10 (45,379,634..45406349, complement)	23,624,869
RAB27A	RAB27A, member RAS oncogene family	Chromosome 15, NC_000015.10 (55,202,966..55291188, complement)	23,553,027
CTDSP1	CTD small phosphatase 1	Chromosome 2, NC_000002.12 (218,398,338..218405941)	28,272,711
PIM1	Pim-1 proto-oncogene, serine/threonine kinase	Chromosome 6, NC_000006.12 (37,170,146..37175428)	27,088,547
TEAD1	TEA domain transcription factor 1	Chromosome 11, NC_000011.10 (12,674,422..12944737)	24,954,504
MAPK14	Mitogen-activated protein kinase 14	Chromosome 6, NC_000006.12 (36,027,635..36122964)	24,954,504
SERP1	Stress associated endoplasmic reticulum protein 1	Chromosome 3, NC_000003.12 (150,541,993..150603177, complement)	24,954,504
R-RAS	RAS related	Chromosome 19, NC_000019.10 (49,635,292..49640143, complement)	24,861,879
N-RAS	NRAS proto-oncogene, GTPase	Chromosome 1, NC_000001.11 (114,704,464..114716894, complement)	22,558,405; 24,861,879
STAT3	Signal transducer and activator of transcription 3	Chromosome 17, NC_000017.11 (42,313,324..42388505, complement)	28,791,348; 23,636,127;
AURKA	Aurora kinase A	Chromosome 20, NC_000020.11 (56,369,389..56392337, complement)	28,393,219; 28,242,198

### eIF4A3 promotes circMMP9 expression

**Fig. 6:**
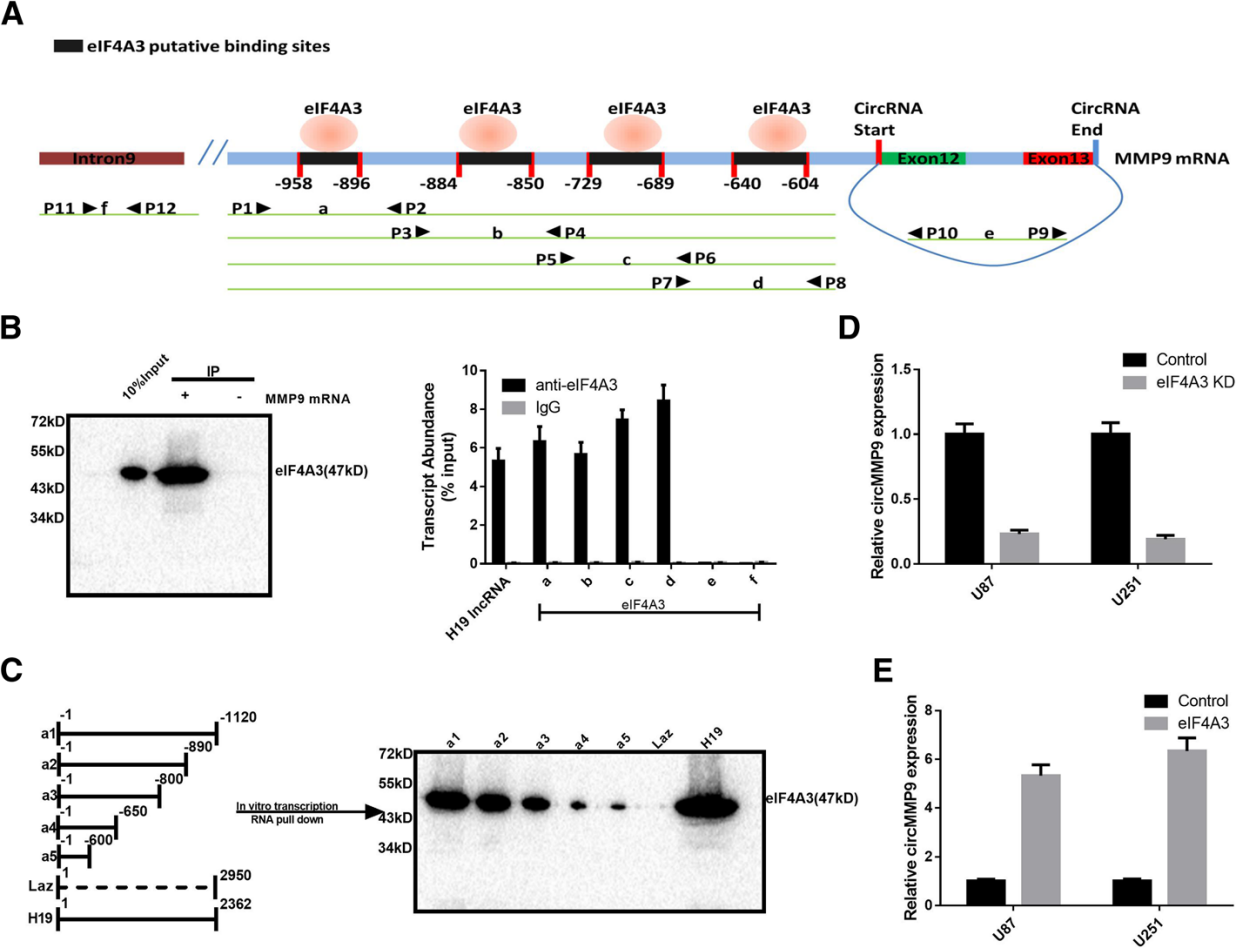
eIF4A3 regulates circMMP9 expression. **a** The binding sites of eIF4A3 were predicted in the upstream region of the MMP9 mRNA transcript using the circRNA interactome. **b** The RIP assay was performed to verify eIF4A3 binding with MMP9 mRNA. H19 lncRNA was used as the positive control, while intron 11 of MMP9 was used as the negative control. qRT-PCR was used to detect the transcript abundance relative to input. **c** A schematic diagram of 5 RNA constructs, which are truncated to varying degrees (a1-a5) and contain the eIF4A3 binding sites, is shown. Laz was a non-sense sequence used as the negative control, and H19 was used as the positive control. The RNA pull-down assay was performed to analyze the interaction between eIF4A3 and MMP9 mRNA (a1-a5). **d** U87 and U251 cells were transfected with control or eIF4A3 knockdown plasmid, and circMMP9 expression was detected by qRT-PCR. **e** U87 and U251 cells were transfected with control or eIF4A3 overexpression plasmid, and circMMP9 expression was measured by qRT-PCR

Using a bioinformatics method (https://circinteractome.nia.nih.gov/index.html), we found that four binding sites for eIF4A3 are present in the upstream region of the circMMP9 mRNA transcript (Fig. 6a). The data from an RIP (RNA binding protein immunoprecipitation) assay using anti-eIF4A3 antibody indicated that eIF4A3 can bind with MMP9 mRNA through the four putative binding sites, which we named a, b, c and d, but not circMMP9 (we named e) in the corresponding RNA-protein complex (Fig. 6b). Next, we constructed 5 RNA transcripts that contained different MMP9 sequences (upstream of circMMP9), and the RNA pull-down assay was performed. The results revealed that the sequence located upstream of circMMP9, which contains eIF4A3 binding sites, is important for the interaction between eIF4A3 and MMP9 mRNA (Fig. 6c). We then knocked down the expression of eIF4A3 and found a reduction in circMMP9 expression (Fig. 6d), while overexpression of eIF4A3 increased circMMP9 expression (Fig. 6e).

### circMMP9 enhances GBM growth in vivo

**Fig. 7:**
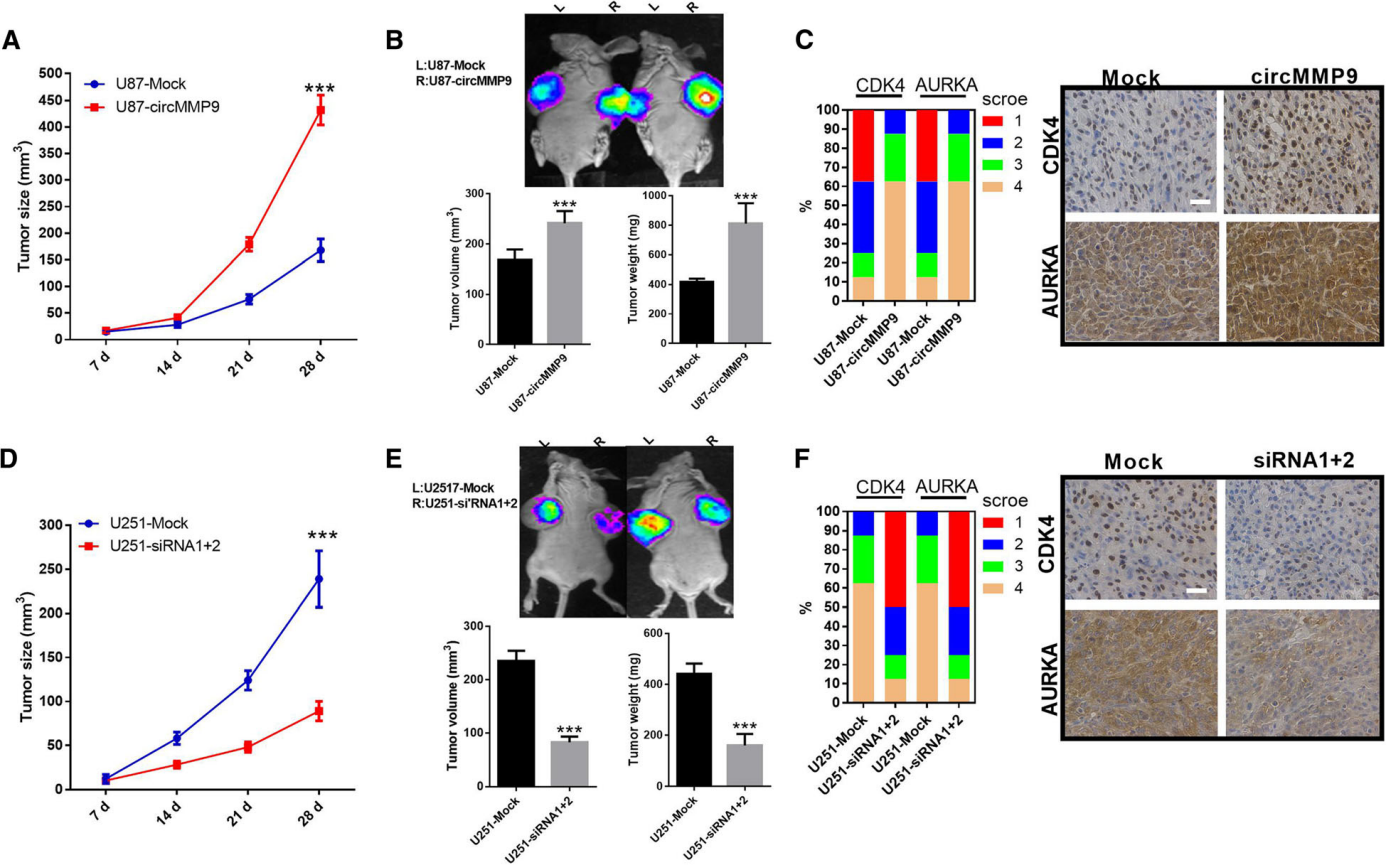
circMMP9 enhances GBM growth in vivo. **a-b** U87 cells were transfected with mock or circMMP9 plasmid. The stable circMMP9 overexpression U87 cells (2 × 10^6^ cells) were injected into nude mice, and tumors were allowed to develop for 7, 14, 21 and 28 days. Representative images of the mice are shown, and the tumor size, volume and weight were determined (***P < 0.001). **c** CDK4 and AURKA expression was detected via IHC. **d-e** Stable circMMP9-knockdown U251 cells (2 × 10^6^ cells) were injected into nude mice, and tumors were allowed to grow for 7, 14, 21 and 28 days. Representative images of the mice are shown, and the tumor size, volume and weight were determined (***P < 0.001). **f** CDK4 and AURKA expression was detected via IHC

In vivo, U87 cells were transfected with the mock or circMMP9 plasmid. U251 cells were transfected with mock or circMMP9 siRNA1 + 2. The stable transfected U87 and U251 cells (2 × 10^6^ cells) were injected in nude mice, and tumors were allowed to grow for 7, 14, 21 and 28 days. The results indicated that the overexpression of circMMP9 generated an outstanding increase in the rate of xenograft subcutaneous tumor growth (Fig. 7a-b). IHC assay data also showed that the overexpression of circMMP9 upregulated CDK4 and AURKA expression (Fig. 7c). We also found that the silencing of circMMP9 generated an marked decrease in the rate of xenograft subcutaneous tumor growth (Fig. 7d-e). The IHC assay data also showed that the silencing of circMMP9 downregulated CDK4 and AURKA expression (Fig. 7f).

## Discussion

Previous research has demonstrated that GBM is challenging for the neurosurgeon due to its rapid proliferation and infiltrative growth, leading to invasion into normal brain tissue and resulting in incomplete resection and recurrence [26]. Therefore, it is important to study the high proliferation and invasion of GBM. Previous studies have shown that circRNA cZNF292 participates in human glioma tube formation [27]; hsa_circ_0046701 promotes glioma carcinogenesis through the miR-142-3p target ITGB8 [28], and circRNA FBXW7 inhibits glioma tumorigenesis [29]. In the present study, we explored the transcriptome of GBM to identify new targets. As a result, we found a novel circRNA, circMMP9, that may serve as an oncogene in GBM. These results further support the pivotal role of circRNAs in GBM. Additionally, we found that CDK4 and AURKA are target genes of the circMMP9/miR-124 axis. These data highlight a novel oncogenic function of circRNA in glioblastoma tumorigenesis. However, there are limitations in our present study. First, only 3 paired GBM tissues were subjected to microarray analysis. Second, only 18 GBM tumor samples were analyzed by PCR for confirmation. In future studies, we need to assess more GBM tissues to confirm the oncogenic role of circMMP9.

Previous research has indicated that eIF4A3 is an important component of RNA splicing [30]. In this study, we found that eIF4A3 could bind to the upstream region of the circMMP9 mRNA transcript and regulate its expression. Therefore, we concluded that eIF4A3 can induce circMMP9 cyclic formation. Of course, further evidence is required to conclude that eIF4A3 can induce circular RNA formation.

In conclusion, our study can be summarized by the following major findings: 1. We identified a novel circular RNA, circMMP9, that acts as an oncogene and promotes GBM proliferation, migration and invasion. 2. We verified that circMMP9 was generated from exons 12 and 13 of MMP9 mRNA. 3. We suggested that circMMP9 can directly bind to miR-124 and regulate its expression. 4. We demonstrated that CDK4 and AURKA are target genes of the circMMP9/miR-124 axis in GBM cells. 5. We confirmed that eIF4A3 induced circMMP9 cyclization and increased circMMP9 expression. Therefore, our study provides a solid basis to develop a better understanding of GBM pathology and identify potential therapeutic drug targets for the treatment of GBM.

## Additional files

Additional file 1: **Table S1.** Primers used in this study. (DOCX 15 kb)

Additional file 2: **Figure S1.** Top 30 gene ontology (GO) enrichment and pathway enrichment results and identification of circMMP9. (A) GO analysis indicated the top 30 Enrichment Score results. The GO domains included biological process, cellular components and molecular function. The top 30 GO enrichment results are shown. (B) Pathway enrichment analysis demonstrated the significant pathways of differentially expressed circRNAs. The top 30 enrichment pathways are shown. (C) cDNA and gDNA expression was measured by PCR using divergent and convergent primers in 3 GBM tissues, U87 cells and U251 cells. (D) circMMP9 and MMP9 mRNA expression was analyzed by qRT-PCR using RNA from U87 and U251 cells treated with RNase R (***P < 0.001). Supplementary Materials and Methods include FISH and microarray analyses. (DOCX 306 kb)

## Abbreviations

ceRNA: competing endogenous RNA
circRNA: circular RNA
GBM: glioblastoma multiforme
miRNA: microRNA
MMP-9: matrix metallopeptidase 9
siRNA: small interfering RNA
