# Correction to: EIF4A3-induced circular RNA MMP9 (circMMP9) acts as a sponge of miR-124 and promotes glioblastoma multiforme cell tumorigenesis

**DOI:** 10.1186/s12943-020-01271-w

**Published:** 2020-10-30

**Authors:** Renjie Wang, Sai Zhang, Xuyi Chen, Nan Li, Jianwei Li, Ruichao Jia, Yuanqing Pan, Haiqian Liang

**Affiliations:** 1 Institute of Traumatic Brain Injury and Neurology, Characteristic Medical Center of Chinese People’s Armed Police Force, Tianjin, 300162 China; 2 Department of Neurosurgery, Characteristic Medical Center of Chinese People’s Armed Police Force, Tianjin, 300162 China; 3 Department of Basic Medicine, Tianjin Medical College, Tianjin, 300222 China; 4 Chinese Glioma Cooperative Group (CGCG), Tianjin, China

**Correction to: Mol Cancer 17, 166 (2018)**

**https://doi.org/10.1186/s12943-018-0911-0**

Following the publication of the original article [1], authors found out that it contains a duplication error within Figs. 4h and 5b. The correct version of Figs. 4 and 5 are shown below. In addition, the correct version of Fig. 1 and Additional file 1 are also provided in this paper.

**Fig. 1.**
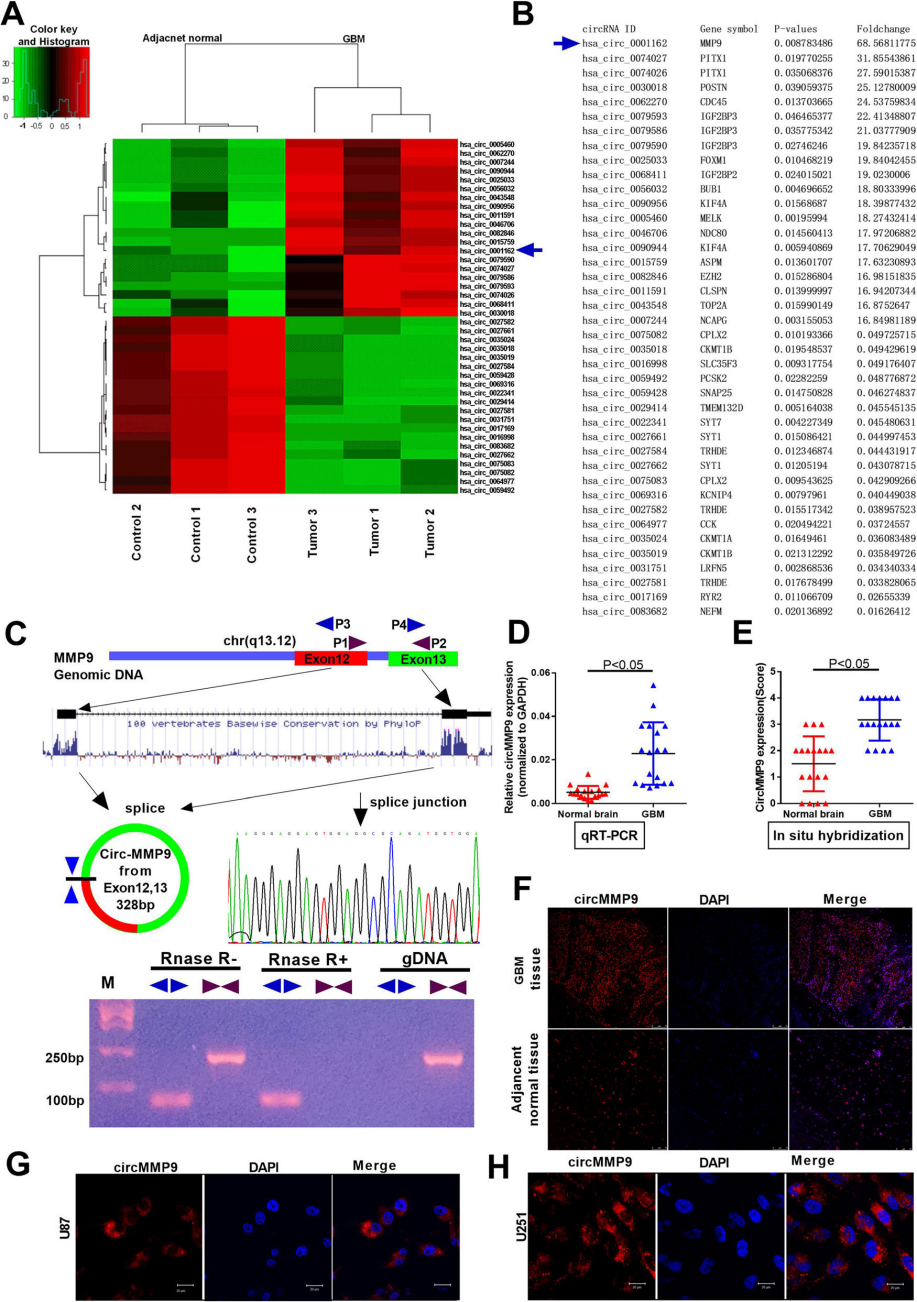
Characterization of circMMP9 in human GBM. **a** Clustered heat map showing tissue-specific circRNAs (top 20 upregulated and downregulated circRNAs), which are displayed on a scale from green (low) to red (high), between three human GBM tissues and adjacent normal tissues. The arrow represents the circRNA (hsa_circ_001162) with the greatest differential expression. **b** Detailed information for the top 20 upregulated and downregulated circRNAs according to the extent. **c** Schematic representation of circMMP9 formation. The splice junction sequence was Sanger sequenced, and the RNAs were detected via PCR. Divergent primers could produce circRNAs in cDNA but not in genomic DNA (gDNA); convergent primers could produce cDNA and gDNA. **d** The expression level of circMMP9 was detected by qRT-PCR in GBM tissues and adjacent normal brain tissues (*n* = 18, *P* < 0.05); GAPDH served as the internal control. **e**-**f** circMMP9 expression was measured using in situ hybridization (FISH) in GBM tissues and adjacent normal brain tissues (*n* = 18, *P* < 0.05). **g**-**h** Confocal FISH was performed to determine the location of circMMP9 in U87 and U251 cells

**Fig. 4:**
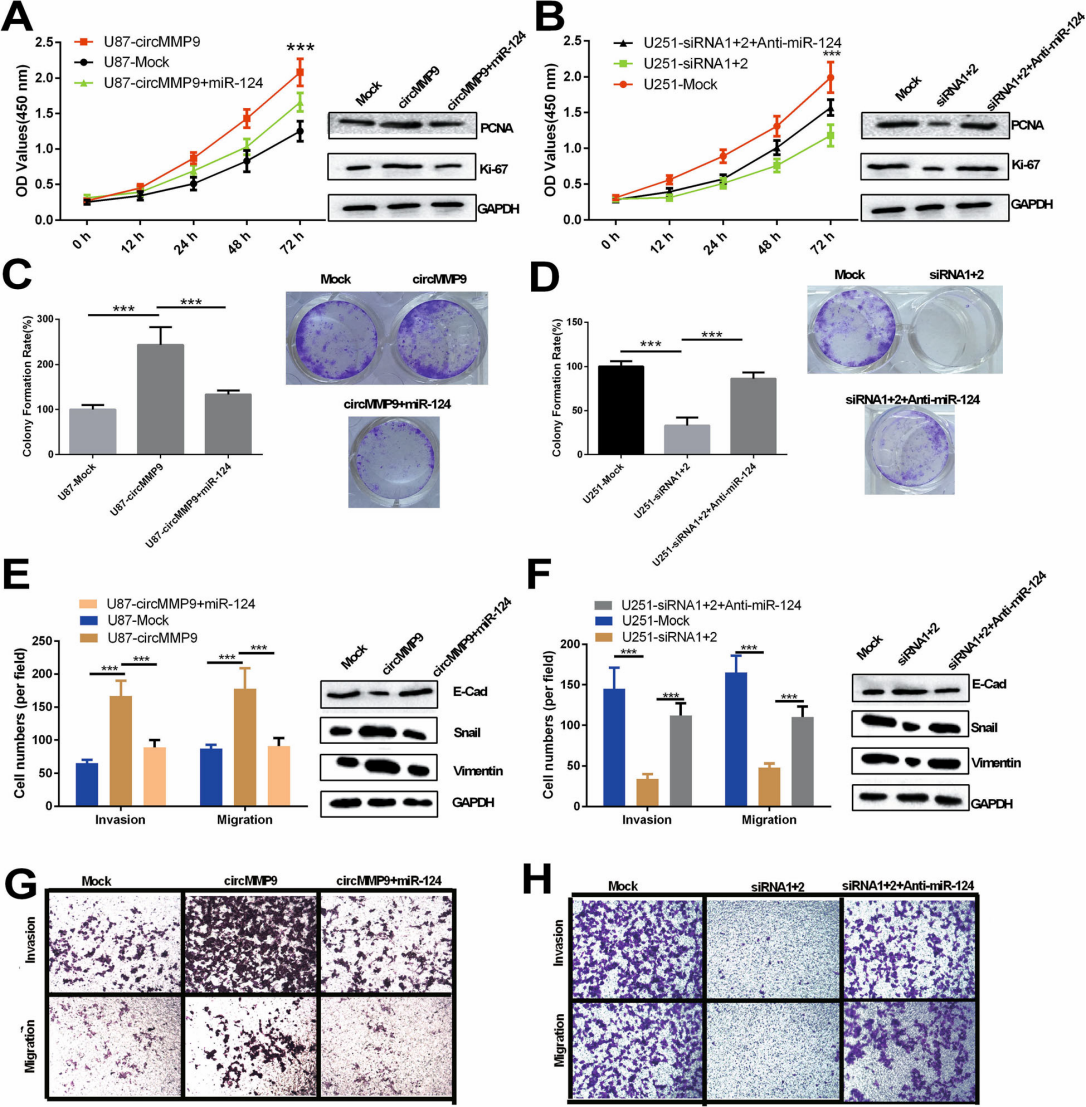
circMMP9 accelerates GBM cell proliferation, migration and invasion by targeting miR-124. **a**-**d** U87 cells were transfected with mock, circMMP9 plasmid, or circMMP9 plasmid and miR-124; U251 cells were transfected with mock, circMMP9 siRNA1 + 2, or circMMP9 siRNA1 + 2 and anti-miR-124. CCK-8 and colony formation assays were performed to assess the proliferation ability of the transfected U87 and U251 cells (***P < 0.001). Western blot assays were used to analyze the protein expression levels of PCNA and Ki67 in transfected U87 and U251 cells. **e**-**h** Transwell assays were performed to evaluate cell migration and invasion abilities (***P < 0.001). Western blot assays were used to analyze the protein expression levels of E-cadherin (E-cad), snail and vimentin in transfected U87 and U251 cells

**Fig. 5:**
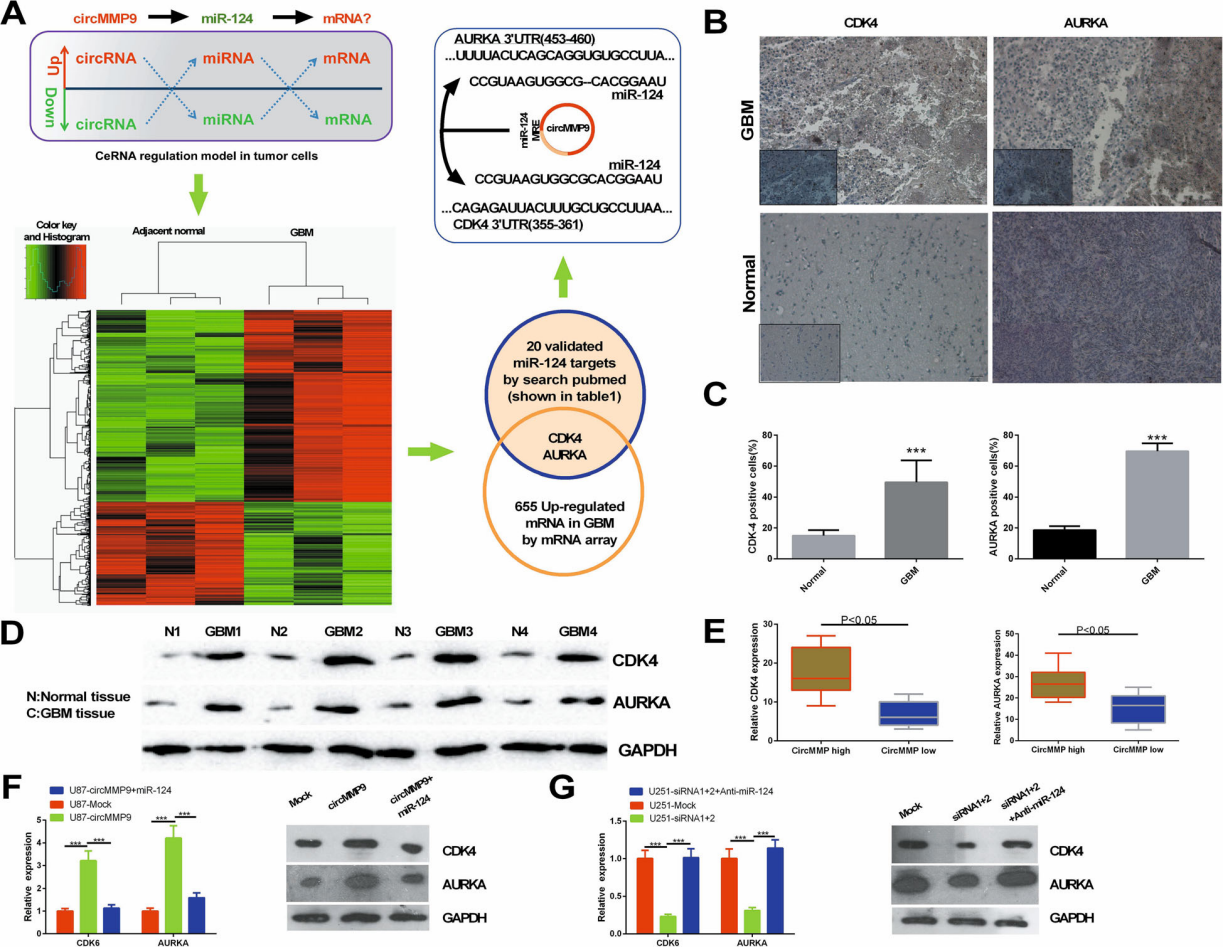
circMMP9 upregulates CDK4 and AURKA via miR-124. **a** Flow chart to screen the targets of miR-124 in GBM. **b** CDK4 and AURKA expression was detected via IHC in GBM tissues and adjacent normal tissues. **c** The cells positive for CDK4 and AURKA were counted (***P < 0.001). **d** The protein expression levels of CDK4 and AURKA were measured by western blot assays in GBM tissues and adjacent normal tissues. **e** CDK4 and AURKA expression was evaluated by qRT-PCR in GBM tissues with high circMMP9 expression or low circMMP9 expression (*P* < 0.05). **f** U87 cells were transfected with mock, circMMP9 plasmid, or circMMP9 plasmid and miR-124. CDK4 and AURKA expression was detected by qRT-PCR and western blot assays (***P < 0.001). **g** U251 cells were transfected with mock, circMMP9 siRNA1 + 2, circMMP9 siRNA1 + 2 or anti-miR-124. CDK4 and AURKA expression was detected by qRT-PCR and western blot assays (***P < 0.001)

## Supplementary information

**Additional file 1 **: **Table S1**. The primers used in this study
