# Supplementary material for: EIF4A3-induced circular RNA MMP9 (circMMP9) acts as a sponge of miR-124 and promotes glioblastoma multiforme cell tumorigenesis
Source: Mol Cancer. 2018 Nov 23;17:166. doi: 10.1186/s12943-018-0911-0 (PMC6260852; doi:10.1186/s12943-018-0911-0)
Supplement: Supplementary file 1 — Table S1. Primers used in this study. (DOCX 15 kb) [file 12943_2018_911_MOESM1_ESM.docx]

Table-2 The primers used in this study:

| circMMP9 | F: 5’-CCA GTT ACA AGT TTA GGG CTG T-3’,  R: 5’- TGT CTC CAT TTG CTT CTT CTT CA-3’ |
| --- | --- |
| miR-124 | F: 5′-GGA CTT TCT TCA TTC ACA CCG-3′,  R: 5′- GAC CAC TGA GGT TAG AGC CA -3′ |
| U6 | F: 5′-CTC GCT TCG GCA GCA CAT ATA CT-3′,  R: 5′- ACG CTT CAC GAA TTT GCG TGT C-3′ |
| CDK4 | F: 5’-AGT GTG AGA GTC CCC AAT GG-3’,  R: 5’- CCT TGA TCT CCC GGT CAG TT-3’ |
| AURK | F: 5’-AGC GGT GGG ATG AGT ACT TT -3’,  R: 5’- TGG TAG TCG TTG TTG TTG GC -3’ |
| GAPDH | F: 5’-GCC ATC ACA GCA ACA CAG AA-3’,  R: 5’-GCC ATA CCA GTA AGC TTG CC-3’ |
| Divergent primers | F: 5’-CCA GTT ACA AGT TTA GGG CTG T-3’,  R: 5’-TGT CTC CAT TTG CTT CTT CTT CA-3’ |
| Convergent primers | F: 5’-AAG TGT CTA GGT CGG GTG TG-3’,  R: 5’- GGT TCA CTG CAG CCC CTA TA-3’ |
