# Supplementary material for: EIF4A3-induced circular RNA MMP9 (circMMP9) acts as a sponge of miR-124 and promotes glioblastoma multiforme cell tumorigenesis
Source: Mol Cancer. 2018 Nov 23;17:166. doi: 10.1186/s12943-018-0911-0 (PMC6260852; doi:10.1186/s12943-018-0911-0)
Supplement: Supplementary file 2 — Figure S1. Top 30 gene ontology (GO) enrichment and pathway enrichment results and identification of circMMP9. (A) GO analysis indicated the top 30 Enrichment Score results. The GO domains included biological process, cellular components and molecular function. The top 30 GO enrichment results are shown. (B) Pathway enrichment analysis demonstrated the significant pathways of differentially expressed circRNAs. The top 30 enrichment pathways are shown. (C) cDNA and gDNA expression was measured by PCR using divergent and convergent primers in 3 GBM tissues, U87 cells and U251 cells. (D) circMMP9 and MMP9 mRNA expression was analyzed by qRT-PCR using RNA from U87 and U251 cells treated with RNase R (***P < 0.001). Supplementary Materials and Methods include FISH and microarray analyses. (DOCX 306 kb) [file 12943_2018_911_MOESM2_ESM.docx]

**Supplementary Figures**


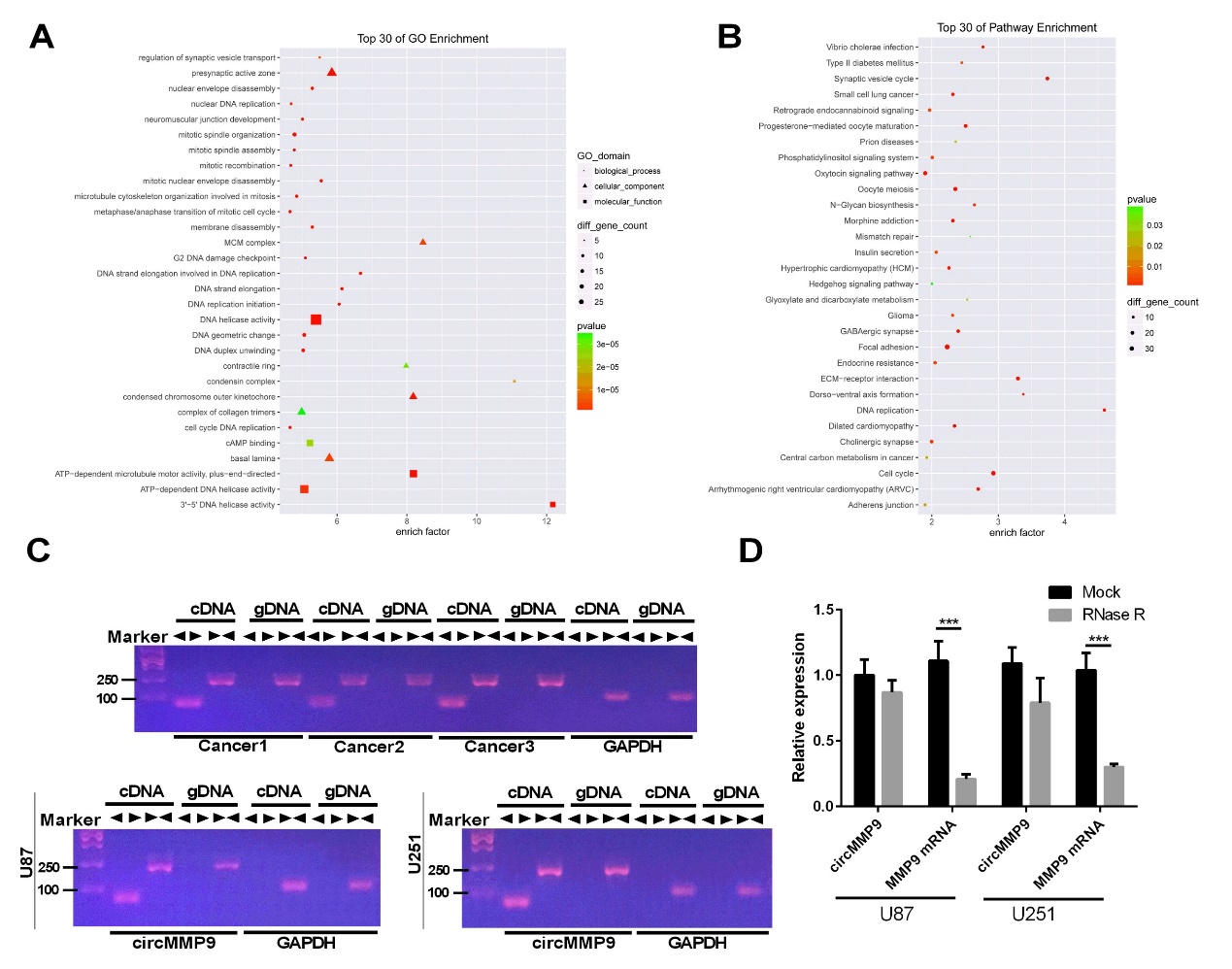


**Figure S1. Top 30 of gene ontology (Go) enrichment and pathway enrichment, and identification of circMMP9.** (**A**) GO analysis indicated the top 30 counts of Enrichment Score. the GO domain included biological process, cellular components and molecular function. Top 30 of Go enrichment was shown. (**B**) Pathway enrichment analysis demonstrated the significant pathways of differentially expressed circRNAs. Top 30 of pathway enrichment was shown. (**C**) cDNA and gDNA expressions were measured by PCR assay by using divergent and convergent primers in 3 GBM tissues, U87 cells and U251 cells, respectively. (**D**) circMMP9 and MMP9 mRNA expressions were analyzed by qRT-PCR assay the RNAs from U87 and U251 cells treated with RNase R (****P* < 0.001).

**Supplementary Materials and Methods**

*Cell culture*

Normal human astrocytes (NHA) cells and GBM cells (U251, SHG44, A172, SNB19 and U87) were purchased from the American Type Culture Collection (ATCC; Manassas, Virginia, USA). All cells were maintained in the sterile culture bottle including RPMI-1640 Medium (HyClone, Cat. No. SH30027), 10% fetal bovine serum (FBS, Cat. 10082-147, Gibco/Life Technologies, Norwalk, Connecticut), 100 U/mL penicillin and 100 U/mL streptomycin (Invitrogen, Carlsbad, CA, Cat. No. 15140-12) at 37 ℃ and 5% CO_2_ saturation.

*siRNA interference*

Control siRNAs (mock) and siRNAs for circMMP9 were obtained from GenePharma Co., Ltd (Shanghai, China). One day before transfection, U251 cells (2×10^4^ cells/well) were seeded into 6-well plates with 2 mL of medium and incubated overnight. Next day, cells were transfected with 50 μM mock, circMMP9‑siRNA1 and circMMP9‑siRNA2 using Lipofectamine 3000 (Invitrogen; Thermo Fisher Scientific, Inc.) according to the manufacturer’s instructions.

*MiRNA transfection*

The miR-124 mimics, miR-124 inhibitor (anti-miR-124) and miRNA controls (mock) were purchased from Shanghai GeneChem Co., Ltd. (Shanghai, China). The sequences of the miR-124 mimics were 5’-UAA GGC ACG CGG UGA AUG CC-3’ and 5’-CAU UCA CCG CGU GCC UUA UU-3’; the sequences of the anti-miR-124 were 5’-GGC AUU CAC CGC GUG CCU UA-3’; the sequences of mock were 5’-UUC UCC GAA CGU GUC ACG UTT-3’. U87 and U251 cells (1 × 10^5^ cells/well) were seeded in six-well plates overnight at 37 ℃ and 5% CO_2_ saturation. U87 cells were transfected with miR-124 and mock; U251 cells were transfected with anti-miR-124 and mock using Lipofectamine 3000 reagent (Invitrogen; Thermo Fisher Scientific, Inc.) according to the manufacturer’s protocol.

*Plasmid construct and transfection*

To construct pcDNA3.1-circMMP9 expression vector, the complete sequence of human circMMP9 gene was synthesized and inserted into a pcDNA3.1 (+) vector (GenePharma, Shanghai, China). U87 cells (5×10^5^ cells) were seeded into 6-well plates and incubated overnight. Then, cells were transfected with pcDNA3.1 (mock) and circMMP9 plasmid using Lipofectamine 3000 (Invitrogen; Thermo Fisher Scientific, Inc.) and Opti-MEM reduced serum medium (Life Technologies) following the manufacturer's protocol. DNA fragments were synthesized for eIF4A3 shRNA and cloned the shRNA into human U6 promoter-containing pBluescript SK (+) plasmid (pU6). Then subclone the U6-shRNA cassettes into a vector (19, 20). U87 and U251 cells (5×105 cells) were seeded into 6-well plates and transfected with mock and eIF4A3 KD plasmids by using Lipofectamine 3000 (Invitrogen; Thermo Fisher Scientific, Inc.) following the manufacturer's protocol.

*Polymerase chain reaction (PCR) assay*

PCR assay was performed by using the Thermal Cycler (S1000, Bio-Rad). The PCR mixtures were shown as follows: Ex Taq DNA Polymerase (1.25 U), 10 × Ex Taq buffer (5μL), dNTPs (4μL, 1 mM each), each primer (0.2 μM) and cDNA template (5 ng). The reaction process was as follows: 94 ℃ for 4 mins, followed by 94 ℃ for 40 s, 658 ℃ for 30 s, 72 ℃ for 1 min for 30 cycles, with a final step for 5 min at 72 ℃. PCR products were detected by using electrophoresis in 1× TAE buffer on a 1.0% agarose gel.

*Western blot assay*

Cells were lysed with RIPA lysis buffer (cat# 89901; Thermo Scientific, Rockford, IL USA)), and protein concentration was measured with a BCA Protein Assay kit (Thermo Fisher Scientific., Rockford, IL, USA). Total proteins (30 μg) were separated, transferred onto polyvinylidene difluoride (PVDF) membranes (Millipore, Billerica, MA, USA), blocked in 5% skim milk (BD Biosciences), incubated with primary antibody overnight at 4°C and then incubated with secondary antibody. the results were obtained by using Enhanced chemiluminescence (ECL) (Amersham Biosciences, Piscataway, New Jersey, USA) and Quantity One V4.6.2 software (Bio-Rad, USA). Primary antibodies used in this study included anti-PCNA (1: 1500 dilution, Abcam, ab29), anti-Ki-67 (1: 1000 dilution, Abcam, ab16667), anti-E-cadherin (1: 1500 dilution, Abcam, ab1416), anti-snail (1: 2000 dilution, Abcam, ab53519), anti-vimentin (1: 2000 dilution, Abcam, ab137321), anti-CDK4 (1: 1500 dilution, Abcam, ab137675), and anti-AURKA (1: 1000 dilution, Abcam, ab13824), and anti-GAPDH (1: 4000 dilution, Abcam, ab9385).

*Immunohistochemistry (IHC) assay*

Immunohistochemistry was performed on 4 µm-formalin-fixed paraffin-embedded tissue sections to detect CDK4 and AURKA expressions. The sections were incubated with anti-CDK4 (1: 50 dilution, Abcam, ab137675), and anti-AURKA (1: 50 dilution, Abcam, ab13824) overnight at 4℃. Immunohistochemical stains were completed using an automated immunostainer (Ventana Medical Systems, Tucson, AZ).

*Luciferase reporter assay*

The putative binding sites of miR-124 on the circMMP9 wild-type (WT) or mutated 3’-UTRs were confirmed (<http://regrna.mbc.nctu.edu.tw/> and www.microRNA.org). Plasmid of circMMP9 (pGMcircMMP9-Lu) was constructed as previously described. After treated with miR-124 for 24 h, U87 cells were cultured and co-transfected with miR-124 or scramble and circMMP9 or vector using Lipofectamine 3000 (Invitrogen; Thermo Fisher Scientific, Inc.) according to the protocol of manufacturer. Then the relative luciferase activity of circMMP9 was measured by a dual luciferase reporter assay kit (Promega) according to the protocol of manufacturer. Each assay was carried out in triplicate and repeated at least three times.

*Fluorescence in situ hybridization (FISH) assay*

FISH was performed by using the probe of circMMP9 and miR-124. PCR products were generated by using the primers of the back-splice region in circMMP9, and then labeled with biotin-labelled RNA probes by using T7 RNA polymerase (Cat. No. M0251L) and Biotin RNA labelling mix (cat. no. 11685597910) according to the protocol of manufacturer. Cells were hybridized by using hybridization buffer (Ultrahyb, Ambion, cat. no. AM8670) including biotin-labelled RNA probes of circMMP9 at 60 ° C overnight. Next day, cells were treated with dyLight 549-conjugated antibody (cat. 711-506-152) and 4,6-diamidino-2-phenylindole (DAPI; Cat# H-1200). The images were obtained by using the Leica DMRXA fluorescence microscope.

*Observation of cell morphology*

U87 and U251 cells (1 × 10^5^ cells/well) were seeded in 6-well plates and incubated with RPMI-1640 medium (HyClone, Cat. No. SH30027) including 10% FBS (Cat. 10082-147) at 37 ℃ and 5% CO2 saturation. Next day, cell morphology was observed by using an inverted microscope (Olympus IX71, Japan).

*Cell Counting Kit-8 (CCK-8) assay*

The treated U87 and U251 cells (2×10^3^ cells/well) were seeded into a 96-well plate and incubated with complete medium at 37 °C for 12, 24, 48, and 72 hrs. Then 15 µl of CCK-8 solution (Dojindo Laboratories, Kumamoto, Japan) was added into each well. After 3 hrs, the absorbance was detected by using a microplate reader at 450 nm.

*Colony formation assay*

The treated U87 and U251 cells were seeded into 6-well plate and incubated with complete medium at 37 °C. After 2 week, the cells were fixed with 4% paraformaldehyde (cat. # 158127-500G) and stained with 2% crystal violet (Cat. # 61135). The images were obtained and the number of colonies was counted.

*Transwell assays*

For migration assay, the treated U87 or U251 cells (1 × 10^5^ cells) in 200 ml of RPMI 1640 medium without FBS were seeded in the upper part of each transwell chamber (pore size, 8 μm; Corning). For the invasion assay, the treated U87 or U251 cells (1 × 10^5^ cells) in 200 ml of RPMI 1640 medium without FBS were seeded in the upper chamber of each insert, which was coated with 50 μl of 2 mg/ml Matrigel growth factor. 600 μl of RPMI 1640 with 20% FBS was added to the lower part of the chamber. After 24 hrs, the upper chamber of insert was cleaned, the membranes were fixed with 4% paraformaldehyde (cat. # 158127-500G) for 10 mins, and stained with 2% crystal violet (Cat. # 61135) for 15 mins. The migrated or invasive cells were counted from five random fields using a light microscope.

**Table S1: clinical patients’ information.**

| **ID** | **Sex** | **Year** | **Grade** | **MGMT** | **GFAP** | **Olig-2** | **EMA** | **Ki-67** | **Vimentin** | **NeuN** | **NF** | **CK** | **S-100** | **P53** | **MMP-9** | **PTEN** | **Syn** | **CDs** | **VEGF** | **IDH1** |
| --- | --- | --- | --- | --- | --- | --- | --- | --- | --- | --- | --- | --- | --- | --- | --- | --- | --- | --- | --- | --- |
| **0089326** | **Male** | **44** | **IV** | **+** | **+++** | **N/A** | **N/A** | **5%-10%** | **N/A** | **N/A** | **N/A** | **N/A** | **N/A** | **N/A** | **N/A** | **N/A** | **N/A** | **N/A** | **N/A** | **N/A** |
| **2907331** | **Male** | **69** | **IV** | **5%** | **+** | **N/A** | **-** | **N/A** | **++** | **N/A** | **N/A** | **N/A** | **N/A** | **+** | **N/A** | **-** | **N/A** | **N/A** | **N/A** | **N/A** |
| **2754818** | **Male** | **64** | **IV** | **+** | **-** | **N/A** | **-** | **>25%** | **N/A** | **N/A** | **-** | **N/A** | **+** | **+++** | **N/A** | **N/A** | **N/A** | **N/A** | **N/A** | **N/A** |
| **1383534** | **Male** | **39** | **IV** | **10%** | **+** | **+** | **+** | **15%** | **+** | **+** | **N/A** | **N/A** | **N/A** | **N/A** | **N/A** | **N/A** | **N/A** | **N/A** | **N/A** | **N/A** |
| **1036693** | **Male** | **60** | **IV** | **5%** | **+** | **N/A** | **+** | **40%** | **+** | **N/A** | **N/A** | **+** | **+** | **N/A** | **N/A** | **N/A** | **N/A** | **CD34-** | **N/A** | **N/A** |
| **1152417** | **Female** | **35** | **IV** | **3%** | **+** | **+** | **-** | **20%** | **N/A** | **N/A** | **-** | **N/A** | **N/A** | **-** | **N/A** | **N/A** | **N/A** | **CD56+** | **N/A** | **-** |
| **1752197** | **Male** | **55** | **IV** | **N/A** | **N/A** | **N/A** | **N/A** | **N/A** | **N/A** | **N/A** | **N/A** | **N/A** | **N/A** | **N/A** | **N/A** | **N/A** | **N/A** | **N/A** | **N/A** | **N/A** |
| **1474282** | **Male** | **47** | **IV** | **-** | **+** | **+** | **N/A** | **70%-80%** | **+** | **+** | **-** | **-** | **N/A** | **N/A** | **N/A** | **N/A** | **N/A** | **CD199+** | **N/A** | **N/A** |
| **1683343** | **Male** | **57** | **IV** | **+** | **+** | **+** | **-** | **20%** | **+** | **N/A** | **N/A** | **-** | **N/A** | **N/A** | **N/A** | **N/A** | **N/A** | **CD34+** | **N/A** | **N/A** |
| **0266417** | **Male** | **43** | **IV** | **+/-** | **+** | **+** | **-** | **60%** | **+** | **N/A** | **N/A** | **-** | **N/A** | **N/A** | **N/A** | **N/A** | **N/A** | **N/A** | **N/A** | **N/A** |
| **1450350** | **Male** | **13** | **IV** | **N/A** | **+** | **+** | **N/A** | **20%** | **+** | **N/A** | **N/A** | **N/A** | **+** | **N/A** | **N/A** | **N/A** | **N/A** | **N/A** | **N/A** | **N/A** |
| **2218546** | **Female** | **62** | **IV** | **-** | **+** | **+** | **-** | **20%-30%** | **N/A** | **N/A** | **N/A** | **N/A** | **N/A** | **20%-30%** | **N/A** | **N/A** | **N/A** | **N/A** | **N/A** | **N/A** |
| **1044951** | **Male** | **44** | **IV** | **-** | **+** | **N/A** | **-** | **15%** | **+++** | **N/A** | **N/A** | **-** | **+++** | **+** | **N/A** | **+** | **N/A** | **N/A** | **N/A** | **N/A** |
| **1287563** | **Male** | **74** | **IV** | **+** | **+** | **+** | **+** | **30%-40%** | **N/A** | **N/A** | **N/A** | **N/A** | **+** | **1%** | **+** | **N/A** | **N/A** | **N/A** | **+** | **N/A** |
| **2744946** | **Female** | **42** | **IV** | **5%** | **+** | **N/A** | **-** | **>40%** | **+** | **N/A** | **N/A** | **N/A** | **-** | **N/A** | **N/A** | **-** | **N/A** | **CD68-** | **N/A** | **N/A** |
| **2739745** | **Female** | **60** | **IV** | **15%** | **+++** | **N/A** | **-** | **20%** | **++** | **N/A** | **N/A** | **N/A** | **+** | **+++** | **N/A** | **-** | **+** | **CD99+** | **N/A** | **N/A** |
| **1722707** | **Male** | **50** | **IV** | **70%-80%** | **+** | **+** | **-** | **15%-20%** | **+** | **-** | **N/A** | **N/A** | **+** | **-** | **N/A** | **N/A** | **N/A** | **CD56+CD99-** | **N/A** | **N/A** |
| **0547181** | **Male** | **40** | **IV** | **-** | **+** | **+** | **N/A** | **15%** | **N/A** | **N/A** | **N/A** | **N/A** | **N/A** | **N/A** | **N/A** | **N/A** | **N/A** | **N/A** | **N/A** | **N/A** |
