# Supplementary material for: Correction to: EIF4A3-induced circular RNA MMP9 (circMMP9) acts as a sponge of miR-124 and promotes glioblastoma multiforme cell tumorigenesis
Source: Mol Cancer. 2020 Oct 30;19:153. doi: 10.1186/s12943-020-01271-w (PMC7597068; doi:10.1186/s12943-020-01271-w)
Supplement: Supplementary file 1 — Additional file 1 : Table S1. The primers used in this study [file 12943_2020_1271_MOESM1_ESM.docx]

Additional file 1

Table S1 The primers used in this study:

| miR-124 | F: 5′- ACACTCCAGCTGGGTAAGGCACGCGGTGA -3′,  R: 5′- TGGTGTCGTGGAGTCG -3′ |
| --- | --- |
| U6 | F: 5′-CTCGCTTCGGCAGCACATATACT-3′,  R: 5′-ACGCTTCACGAATTTGCGTGTC-3′ |
| CDK4 | F: 5’-GAGGGGGCCTCTCTAGCTT-3’,  R: 5’-CACGGGTGTAAGTGCCATCT-3’ |
| AURKA | F: 5’-GGATATCTCAGTGGCGGACG-3’,  R: 5’-TGGTTGCCTGCAATTGCTTC-3’ |
| GAPDH  Divergent primers | F: 5’-GTATTGGGCGCCTGGTCACC-3’,  R: 5’-CGGCTGGCGACGCAAAAGAA-3’ |
| GAPDH  Convergent primers | F: 5’-TTCTTTTGCGTCGCCAGCCG-3’,  R: 5’-GGTGACCAGGCGCCCAATAC-3’ |
| CircMMP9  Divergent primers | F: 5’-GAGCCAGTTTGCCGGATA-3’,  R: 5’-CTCCGGGGATCCACCATCT-3’ |
| CircMMP9  Convergent primers | F: 5’-CGACGTCTTCCAGTACCGAG -3’,  R: 5’-TTGTATCCGGCAAACTGGCT-3’ |
